# Mindfulness and Psychosocial Symptoms in People with Cancer: Testing Rumination and Experiential Avoidance as Mediators, and Sex as a Moderator

**DOI:** 10.1177/27536130251367051

**Published:** 2025-08-08

**Authors:** Hanna S. W. Conradi, Tina Nguyen, Oluwaseyi A. Lawal, Linda E. Carlson

**Affiliations:** 1 Department of Psychology, 2129 University of Calgary, Calgary, AB, Canada; 2 Department of Oncology, 2129 University of Calgary, Calgary, AB, Canada

**Keywords:** psychosocial oncology, mindfulness-based interventions, depression, anxiety, fear of cancer recurrence, fatigue

## Abstract

**Objectives:**

While Mindfulness-Based Interventions (MBIs) are evidenced to reduce common psychosocial symptoms experienced by people with cancer (PWC), few studies have tested their mechanisms. Additionally, studies have yet to assess sex assigned at birth as a moderator of the relationship between mindfulness and psychosocial symptoms through specified mediators. This study (1) explored the mediating role of rumination and experiential avoidance (EA) in the relationship between mindfulness and a range of psychosocial symptoms and (2) tested sex as a moderator of the mediation models.

**Methods:**

This cross-sectional study assessed baseline data from 134 participants recruited for a mindfulness app clinical trial. Validated patient reported outcome measures of trait mindfulness, rumination, experiential avoidance, depression, anxiety, FCR, and fatigue were collected. Structural Equation Modelling was employed in R.

**Results:**

Rumination was a significant partial mediator between mindfulness, depression and anxiety and FCR, but not fatigue. EA acted as a weak mediator from mindfulness to FCR only. Subgroup analyses found that rumination may be more important for females than males in the relationship between mindfulness and depression and anxiety.

**Conclusions:**

Rumination may be a stronger mediator than EA for anxiety, depression and FCR, and this may be particularly important for females. Findings may help MBI researchers and developers target potentially relevant mediators to maximize robust study design and intervention efficacy.

## Introduction

Psychosocial symptoms, such as depression, anxiety, and fatigue, are more prevalent in people with cancer (PWC) (ie, those previously diagnosed with cancer) than the general population; PWC also suffer significant and sometimes debilitating fear of cancer recurrence or progression. Interventions that target these symptoms can reduce symptom burden and improve quality of life.^1^ Depression is up to twice as high in PWC compared to the general population with a long-term prevalence of 17% (ie, 5 to 10 years after receiving a cancer diagnosis).^2-5^ Similarly, 10-30% of PWC experience anxiety disorders at some point after diagnosis, with 21% suffering long-term.^2,4,6-8^ Depression and anxiety are more common in female PWC.^3,8-11^

A specific form of anxiety that is unique to PWC is the fear that cancer will return or progress, referred to as fear of cancer recurrence (FCR).^6^ A recent study reported that 64.3% of PWC experience high to clinical levels of FCR,^12^ and like depression and anxiety, female PWC tend to experience heightened FCR.^13,14^ Fatigue is another highly prevalent symptom, present in up to 99% of PWC during treatment, and is maintained for months or years after treatment in up to 82% of patients.^15,16^ The incidence and severity of fatigue is also higher in females.^17^ Prolonged high levels of depression, anxiety, FCR, and fatigue can reduce quality of life and treatment adherence in PWC.^6^ Therefore, understanding potentially treatment-responsive correlates of lower psychosocial symptoms may inform intervention content and delivery, and thereby improve treatment outcomes in PWC.

Depression, anxiety, FCR, and fatigue are symptoms which tend to be associated with one another in various ways (ie, they cluster).^1,5,6,9,17-25^ For example, anxiety and depression present as a symptom cluster,^26,27^ suggesting that testing them together might help to better understand what mechanisms account for changes in both symptoms simultaneously. Additionally, MBIs reduce FCR, and fatigue concurrently with depression and anxiety,^1^ suggesting that there may be a shared underlying mechanism by which MBIs target each of these symptoms. However, studies have yet to test underlying mechanisms of MBIs accounting for reductions in each of the aforementioned symptoms.

## Mindfulness-Based Interventions (MBIs)

MBIs are typically multi-week, group-based mindfulness training programs modelled on Kabat-Zinn’s Mindfulness-Based Stress Reduction (MBSR) program.^1^ These include Mindfulness-Based Cognitive Therapy and other adaptations. Accumulating evidence indicates that MBIs improve anxiety, depression, FCR, and fatigue in people with various chronic health conditions, including cancer,^28-32^ but the mechanisms by which these programs affect change are uncertain. One mechanism is through increasing levels of mindfulness,^33^ but others have been postulated as well, including ER.^34,35^

### Emotion Regulation and Mindfulness

Emotion Regulation (ER) refers to “attempts to influence which emotions one has, when one has them, and how one experiences or expresses these emotions”.^36(p5)^ People may benefit from mindfulness training by reducing the overuse of maladaptive ER strategies,^1,37^ with “maladaptive” defined as using strategies which are ineffective for the specific context or goal.^38-40^ Some of these maladaptive ER strategies include rumination and experiential avoidance (EA). Rumination is a type of perseverative thinking that involves repetitive negatively-valanced thoughts that maintain or exacerbate uncomfortable emotions, is highly correlated with depression and anxiety, and more common in females and younger adults than males and older people.^41-44^ An example of rumination is replaying an event that elicited uncomfortable emotions over and over in one’s mind, like an embarrassing moment, leading to intensified feelings of sadness and shame. EA entails any behavioural or cognitive evasion of experience that one finds unpleasant or unappealing, and is associated with elevated anxiety, depression, FCR, and fatigue, and is also higher in females than males.^45-47^ An example of behavioral EA is staying home from an important social event out of fear of feeling uncomfortable and embarrassed. Cognitive EA may be the refusal to admit to oneself that staying home was indeed out of fear or anxiety.

People often react with ER strategies automatically without conscious awareness. Therefore, increasing awareness through mindfulness may help to ensure ER responses are more effective and, in turn, adaptive.^33,48^ Mindfulness first requires purposely removing ones attention from engaging in rumination and/or EA, and shifting attention towards present moment experience, often focusing on sensory input.^35^ Mindfulness also requires applying an attitude of acceptance and non-judgment to the present moment, which allows for clearer information gathering about one’s context, including better goal identification.^33^ Gathering relevant contextual information and adequately identifying goals are integral to effective ER.^33,35^

Mindfulness can be considered both as a way of being in the world and as a skill that one learns through practice (eg, meditation) over time.^1,6,49^ Trait mindfulness refers to the former, specifically the degree to which people are mindful in their day to day lives whereas state mindfulness is the degree to which a person is mindful in a given moment, particularly when they are practicing mindfulness meditation. MBIs are thought to work in part by increasing overall *trait* mindfulness through consistent mindfulness meditation practice, which essentially is the act of increasing the capacity and propensity for *states* of present moment awareness through focused attention training.^33^ Formal mindfulness meditation practice changes habitual patterns of thinking and responding and thereby spreads into informal mindfulness in everyday life, leading to overall higher trait mindfulness. When referring to mindfulness throughout this paper, we are referring to measures of trait mindfulness that have been used in both cross-sectional and MBI studies.

In general, self-reported mindfulness correlates negatively with self-reported measures of psychological distress and maladaptive ER in nonclinical samples (ie, of adults and undergraduate students).^50^ Sex assigned at birth (from now referred to as sex) shows consistent correlations with varying levels of mindfulness in non-cancer populations. For example, males report themselves as having higher levels of mindfulness than females.^51^ Given that sex is associated with differing levels of mindfulness, delineating the potential moderating role of sex in the relationship between mindfulness and psychosocial symptoms will help in understanding who may benefit most from mindfulness practice, since it is not a “one size fits all” approach and different MBI strategies may be best suited for particular people.

Previous intervention and cross-sectional studies have found that rumination and EA act as mediators of mindfulness in PWC, supporting theoretical connections between mindfulness and ER.^34,52-54^ Importantly, changes in facets of mindfulness (eg, present focused attention and awareness as well as nonjudgement and observing) precede changes in ER and coping factors during MBIs, suggesting that mindfulness may be directly causing these changes.^34,35^ However, studies have yet to test rumination and EA as mediators to FCR and fatigue. Similarly, sex has well-evidenced associations with psychosocial symptoms, mindfulness, and mediating mechanisms such as rumination and EA, but studies have yet to include sex as a potential moderator of the relationship between mindfulness and psychosocial symptoms *through* rumination and EA. Indeed, because so many mindfulness studies include almost entirely or exclusively female participants, this type of analysis has not been possible. Given the links between sex, psychosocial symptoms, mindfulness, and ER, the next step is to examine precisely how these factors may be interacting with one another.

### Present Study

This cross-sectional study utilized baseline data from a randomized waitlist-controlled clinical trial of a mindfulness-based digital health intervention (DHI) in PWC,^55^ and received ethics approval from the Health Research Ethics Board of Alberta - Cancer Care. The mediating role of ER strategies (rumination and EA) in the relationship between baseline trait mindfulness and psychosocial symptoms of depression, anxiety, FCR, and fatigue was investigated. The moderating role of the demographic characteristic of sex in these mediated relationships was also explored. The purpose of using a cross-sectional design in this study is to provide preliminary insights that can strengthen the rationale and methodological planning of future experimental MBI studies. While cross-sectional mediation is limited in its ability to establish temporal ordering and causality, in this case, there is substantial theoretical and empirical support for the presumed directionality of the variables being modeled and the use of trait mindfulness as a proxy for the impact of MBIs.^33,34^ These findings lend credence to modeling these relationships cross-sectionally. Importantly, this study is the first to examine rumination and EA as mediators between mindfulness and FCR and fatigue, as well as the first to test sex as a moderator of these mediated pathways. By exploring these mechanisms in a cross-sectional framework, we aim to identify promising patterns that can inform the design of more targeted and individualized MBIs. Although we acknowledge the inherent limitations of cross-sectional data, the current findings serve as a theoretically grounded and necessary step toward more robust experimental investigations into the long-term psychosocial impacts of cancer and its treatment.

## Method

### Objectives

The present study conducted a novel investigation in a diverse sample of PWC to cross-sectionally assess:

1. the mediating role of *rumination* in the relationship between mindfulness and psychosocial symptoms of depression and anxiety, FCR, and fatigue
2. the mediating role of *EA* in the relationship between mindfulness and psychosocial symptoms of depression and anxiety, FCR, and fatigue in a diverse sample of PWC
3. explore sex as a moderator of the mediation (see Figure 3 for path diagrams).

### Participants

Participants were PWC interested in participating in the SmartphonE App-based MindfuLnESS (SEAMLESS) study and who had completed primary treatments (eg, surgery, chemotherapy, radiation therapy) for any type and stage of cancer at least 2 weeks prior. Participants suffering from untreated psychiatric disorders or who had previous experience practicing in in-person or app-based mindfulness once a week or more within the last year were excluded. A variety of recruitment methods were utilized to reach diverse populations of PWC and target specific subgroups.

### Recruitment

Participants were recruited through invitation letter mailouts using the Alberta population-based cancer registry, via word of mouth (clinician to patient), through social media posts, as well as using pamphlets and TV screens in waiting areas at the local cancer centre. Sample diversity was maximized by oversampling males and younger people and mailing to postal codes where many people of lower SES, immigrants and racialized groups are known to live (using data from Statistics Canada).

### Procedure and Materials

Interested participants first contacted the study team via toll-free telephone or email. Participants underwent eligibility screening over the phone and if deemed eligible, were emailed a link to the online consent form on the Remote Electronic Data Capture (REDCap) system. After obtaining informed consent, participants were automatically emailed a link to complete the baseline assessment measuring demographic variables, psychosocial outcomes, ER strategies, and mindfulness. Assessments included the following self-report measures:

#### Demographics

Demographic information was collected on a variety of sociocultural factors including age, sex, marital status, income, employment and education level, disability, ethnicity and cultural background. Additionally, disease characteristics such as cancer diagnosis at the time of enrollment, cancer stage, last treatment date and date of birth were extracted from participants’ screening forms.

#### Rumination-Reflection Questionnaire (RRQ)

The RRQ has 25 items assessing mostly past-oriented recurrent thinking about the self often triggered by threats, losses, or injustices to the self. The measure involves a 5-point rating scale where respondents indicate the degree to which statements apply to their experience. Higher scores on the rumination subscale indicate higher levels of rumination.^56^ Internal consistency is good (.90) and validation efforts have shown stability over 10-months as well as convergent validity in general adult and student populations.^56^

#### Acceptance and Action Questionnaire (AAQ)

The AAQ has 16 items and was developed to measure the tendency to negatively evaluate internal experiences: emotions, body sensations, as well as unwillingness to be in contact with such experiences, and the need to control or alter them or the contexts that engender them.^46,57^ Higher scores indicate high EA. The AAQ has evidenced convergent and concurrent validity and internal consistency ranging from a = .74-94.^46,57^ Halfway through data collection, the study team switched to the AAQ short-form 7-item version as per patient partner request. The short-form was developed to improve psychometric properties of the original version and also has demonstrated convergent and concurrent validity with high internal consistency (a = .81-.87) and test-retest reliability (a = .81-.79) in various adult and student populations.^53,58^

#### Mindful Attention and Awareness Scale (MAAS)

The MAAS has 15 items and was developed to measure the presence or absence of attention to and awareness of what is occurring in the present moment. The measure includes a 5-point rating scale by which respondents rate the frequency of their experiences with present moment awareness and attention. Higher scores indicate higher trait mindfulness. The construct and criterion validity has been validated in cancer populations and thoroughly tested in general adult and student populations (Brown & Ryan, 2003; Carlson & Brown, 2005).

#### Fear of Cancer Recurrence or Progression Inventory (FCRI)

The Fear of Cancer Recurrence Inventory (FCRI) has 42 items and was used to measure FCR in 8 subscales: (1) Triggers, (2) Severity, (3) Psychological Distress, (4) Functional Impairment, (5) Insight, (6) Reassurance, and (7) Coping Strategies. Higher scores indicate higher trait mindfulness.^59,60^ High internal consistency (a = .95), temporal stability (r = .89), and construct validity (r = .68-.77) has been demonstrated in a large sample of French-Canadian cancer patients.^59,60^

#### Patient Reported Outcomes Measurement Information System (PROMIS)

PROMIS measures have been standardized across all conditions. PROMIS measures used in the current study include assessments of Anxiety, Depression, and Fatigue Banks (all v1.0). Anxiety items (22) measure self-reported fear (fearfulness, panic), anxious misery (worry, dread), hyperarousal (tension, nervousness, restlessness), and somatic symptoms related to arousal (racing heart, dizziness). Depression items (30) measure self-reported negative mood (sadness, guilt), views of self (self-criticism, worthlessness), and social cognition (loneliness, interpersonal alienation), as well as decreased positive affect and engagement (loss of interest, meaning, and purpose). Fatigue items (54) measure symptoms of fatigue such as feeling “run-down” or “physically drained”. High scores represent higher levels of these symptoms. Construct validity has been demonstrated with moderate to strong correlations with other well-validated measures.^61^

### Statistical Analyses

Preliminary analyses to test for violations in the assumptions of structural equation modeling (SEM) and assess the nature of missing data were conducted. Mean imputation was applied to manage data missing completely at random.^62,63^ To account for normality violations, bootstrapped estimates of the structural models were obtained. SEM was conducted using the program R (version 2024.04.0 + 735, Lavaan package) to test the hypotheses. The Bonferroni correction method was applied and set at 0.003 for all models, resulting in a very conservative definition of “significance”. Mediation models involved mindfulness as an exogenous variable and EA and rumination as endogenous mediating variables that were tested separately in single mediation models. Three psychosocial symptom outcome variables were tested: FCR and fatigue as separate observed variables, and depression and anxiety as a latent cluster variable (See Figures 2 and 3 for path diagrams). The moderation and moderated mediation models involved including sex as a moderator of the direct and indirect paths in each mediation model that had significant indirect and/or direct effects (See Figure 4 for path diagrams). To test the measurement models, model fit indices included the chi-square (χ^2^) test, Comparative Fit Index (CFI), Tucker-Lewis Index (TLI), Root Mean Square Error of Approximation (RMSEA), and Standardized Root Mean Squared Residual (SRMSR) and are reported as supplemental material (Table 1).^64^ To test the structural models, the bootstrapped path coefficients (β) and their corresponding standard errors were examined and z-values tested for statistical significance.

## Results

### Participants

**Figure 1.**
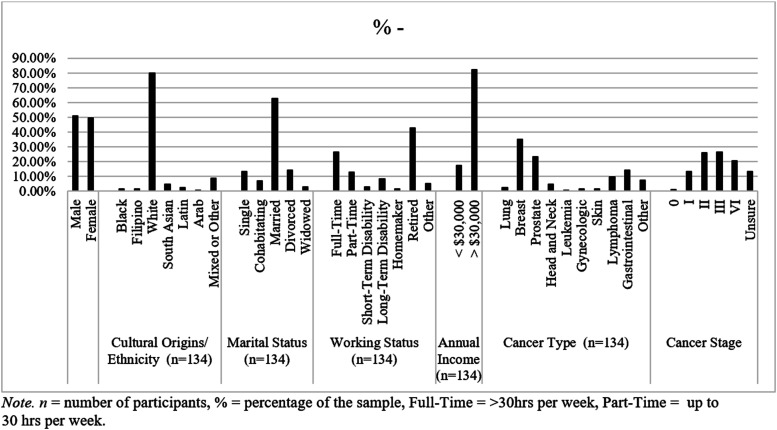
Participant demographics. *Note.* n = number of participants, % = percentage of the sample, full-time = >30 hrs per week, Part-Time = up to 30 hrs per week

At the time of data-extraction, this cross-sectional study had screened 163 participants. The final number eligible, providing consent and completing baseline data collection was n = 134. Despite efforts to target a more ethnically diverse sample, 79.9% of this sample identified as white. Diversity was achieved in sex, with 49.3% female participants, and in income, cancer type and stage, with almost half the sample living with advanced disease (Stages III or IV). The mean age of participants was 60.40 (SD = 13.87) years, and the average education was 15.31 (SD = 5.07) years. 17.2% of participants had an annual income of less than $30,000 (many of these were retired). The minimum time since treatment ranged from 17 days to over 17 years (SD = 860.26). Figure 1 shows the participant demographics for this sample.

### Structural Equation Models (SEM)

**Figure 2.**
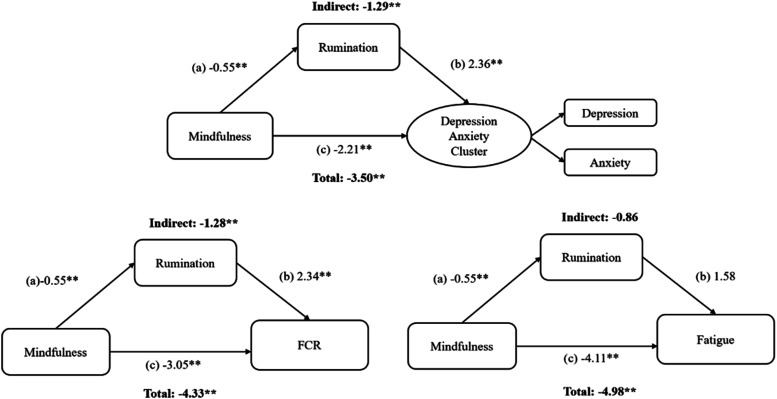
Single mediation models testing rumination: structural equation modeling path diagrams showing rumination as mediator between mindfulness and various psychosocial symptom variables. *Note.* a = direct effect of mindfulness on rumination, b = direct effect of rumination on outcomes, c = direct effect of mindfulness on outcomes, Indirect = indirect effect of mindfulness through rumination on outcomes. Figure represents three separate statistical tests. Significant *P*-values (*P* = .003) are indicated by a double asterisk (**) when below or equal to .001 and a single asterisk when (*) when between .002 and .003. Total = total effect of mindfulness on outcomes through direct and indirect effects

**Figure 3.**
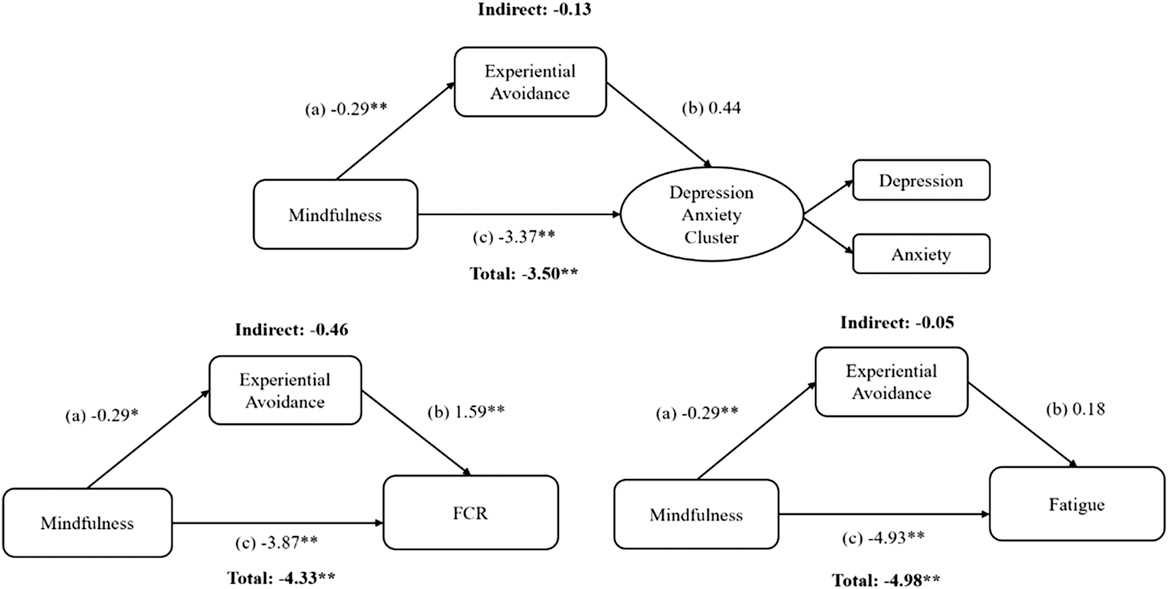
Single mediation models testing experiential avoidance: structural equation modeling path diagrams showing experiential avoidance as mediator between mindfulness and various psychosocial symptom variables. *Note.* a = direct effect of mindfulness on EA, b = direct effect of rumination on outcomes, c = direct effect of mindfulness on outcomes, Indirect = indirect effect of mindfulness through rumination on outcomes. Figure represents three separate statistical tests. Significant *P*-values (*P* = .003) are indicated by a double asterisk (**) when below or equal to .001 and a single asterisk when (*) when between .002 and .003. Total = total effect of mindfulness on outcomes through direct and indirect effects

**Figure 4.**
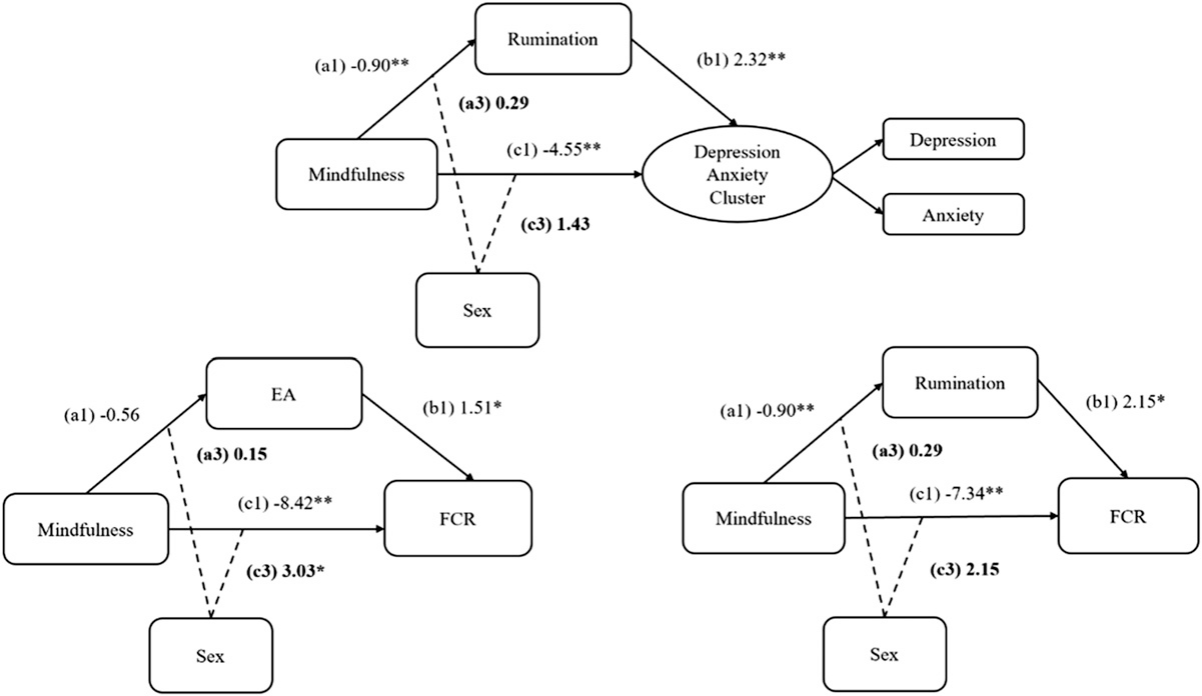
Moderated mediation model testing sex as a moderator: structural equation modeling path diagrams showing sex as moderator of direct and indirect paths between mindfulness, rumination, experiential avoidance, and psychosocial outcomes. *Note.* al = direct effect of mindfulness on rumination or EA, a3 = sex as moderator of indirect path, b1 = direct effect of rumination on outcomes, c1 = direct effect of mindfulness on outcomes, c3 = sex as moderator of direct path. Figure represents three separate statistical tests. Significant *P*-values (*P* = .003) are indicated by a double asterisk (**) when below or equal to .001 and a single asterisk when (*) when between .002 and .003. Total = total effect of mindfulness on outcomes through direct and indirect effects

Table 1 presents the descriptive statistics for the variables being tested in the models. Bootstrapped estimates are reported in Figures 2–4 and in table form as supplemental material (Table 2). A correlation matrix and heatmap is also available in supplemental material (Figure 1). All effect sizes were small to medium and larger for males than females.

**Table 1.** Descriptive Statistics of Patient Reported Outcomes and Sex Differences

Measure	Overall	Male	Female	Range	t-value
	Mean (SD)	Mean (SD)	Mean (SD)	(Min, Max)	
Mindfulness (MAAS)	4.48 (0.98)	4.86 (0.86)	4.08 (0.95)	1.80, 6.00	5.02**
Rumination (RRQ)	3.21 (0.92)	2.80 (0.91)	3.62 (0.74)	1.33, 4.92	−5.70**
Experiential avoidance (AAQ)	0.00 (1.00)	−0.05 (0.95)	0.05 (1.05)	−2.47, 2.71	−0.56
Anxiety (PROMIS)	11.96 (5.39)	10.38 (5.16)	13.59 (5.18)	6.00, 30.00	−3.59**
Depression (PROMIS)	6.73 (3.39)	6.19 (3.19)	7.29 (3.51)	4.00, 16.00	−1.91*
Fatigue (PROMIS)	21.87 (8.41)	20.15 (8.65)	23.65 (7.82)	8.00, 40.00	−2.46*
Fear of cancer recurrence (FCRI)	15.36 (7.27)	13.47 (7.51)	17.31 (6.51)	0.00, 30.00	−3.16**

*Note. M* = mean, SD = standard deviation*, t* = t-value, Significant *P*-values of t-tests (*P* = .05) are indicated by a double asterisk (**) when below .001 and a single asterisk when (*) when below .05, Total n *=* 134.

### Objective 1: Rumination as Mediator Between Mindfulness and Psychosocial Symptoms

#### Depression and Anxiety Cluster

Depression and anxiety significantly loaded onto the cluster variable with standardized loadings of 0.99 and 0.79, respectively. All path coefficients were statistically significant and so were the indirect and total effects (Figure 2). The model accounted for 51% of the variance in the outcome cluster, 99% of the variance in observed anxiety, 62% of the variance in observed depression, and 34% of the variance in rumination.

#### Fear of Cancer Recurrence (FCR)

All paths and indirect and total effects were statistically significant (Figure 2). The model accounted for approximately 40% of the variance in FCR and 34% of the variance in rumination.

#### Fatigue

The path from rumination to fatigue was not significant indicating that rumination did not mediate the relationship between mindfulness and fatigue. This is further supported by the non-significant indirect effect through rumination to fatigue but the significant total effect (Figure 2). Although the indirect effect of rumination on fatigue was not significant, the model still accounted for 36% of the variance in fatigue, likely due to the significant direct effect of mindfulness on fatigue.

### Objective 2: Experiential Avoidance as Mediator Between Mindfulness and Psychosocial Symptoms

#### Depression and Anxiety Cluster

All path coefficients except the b path (ie, from EA to depression and anxiety) were statistically significant (Figure 3). Consistent with this finding, the indirect effect was not significant while the total effect was. The model accounted for 51% of the variance in the outcome cluster, 99% of the variance in observed anxiety, 62% of the variance in observed depression, and 34% of the variance in rumination.

#### Fear of Cancer Recurrence (FCR)

While all direct paths were significant, the a path (ie, from mindfulness to EA) was just at the alpha cut-off suggesting a potentially weaker indirect effect (Figure 3). The direct effect was not significant while the total effect was. The model accounted for approximately 38% of the variance in FCR but only 8% of the variance in EA.

#### Fatigue

Similar to the model above, the path from EA to fatigue was not significant in this model, indicating that EA did not significantly mediate the relationship between mindfulness and fatigue (Figure 3). This is further supported as the indirect path through rumination to fatigue was not significant. The model accounted for approximately 36% of the variance in fatigue and 34% of the variance in rumination.

### Objective 2: Sex as Moderator

Sex was tested as a moderator for all models that had statistically significant indirect effects and/or a and b paths (Figure 4). Model fit indices for all these models were meeting requirements and estimates were reported in supplemental material (Table 1). The effect of the interaction between sex and predictors on direct and indirect paths was only significant in one instance (ie, through the direct path to FCR when controlling for EA), indicating that the influence of sex on mediated relationships among variables was not statistically significant. However, when the direct, indirect, and total effects were tested for significance at different levels of sex in subgroup analyses (ie, male vs female) some differences in *P*-values and effect sizes emerged, are highlighted below (Supplemental Material, Table 2, for estimates).

#### Through Rumination to Depression and Anxiety

The same statistically significant relationships among variables remained when sex was added to the model. The indirect and total effects were significant for both levels of the moderator separately (ie, male vs female), though effect sizes were small. This suggests that mindfulness may work to improve depression and anxiety through reducing rumination for both males and females. The direct effect from mindfulness to depression and anxiety was significant for males but not for females suggesting rumination may act as a full mediator from mindfulness to depression and anxiety for females but not males. There was no statistically significant difference in the mediated effects between males and females. The model accounted for 98% of the variance in anxiety, 63% of the variance in depression, 41% of the variance in rumination and 54% of the variance in depression and anxiety as a cluster.

#### Through Rumination to Fear of Cancer Recurrence (FCR)

The direct, indirect, and total effects remained significant when sex was added to the model. The indirect effects through rumination to FCR were not significant for males or females. There was no statistically significant difference in mediated effect across levels of sex. The direct effect was significant for males but not for females while the total effect was significant for both. The model accounted for 41% of the variance in rumination and 43% of the variance in FCR.

#### Through Experiential Avoidance (EA) to Fear of Cancer Recurrence (FCR)

The path from mindfulness to EA became non-significant when sex was added to the model suggesting that sex accounts for some of the variance previously attributed to the relationship between mindfulness and EA. However, the c and b paths maintained significant path coefficients to FCR with small to medium effect sizes (Figure 4). Results indicate non-significant indirect effects for both males and females. However, the direct and total effects for both males and females were significant meaning that mindfulness was associated with FCR in similar ways for both males and females while controlling for EA. This is further supported by the significant interaction between mindfulness and sex on the direct path to FCR illustrating a significant moderating impact of sex on the direct relationship between mindfulness and FCR. The model accounted for 9% of the variance in EA and 43% of the variance in FCR.

## Discussion

This is the first study to specifically test rumination and EA as mediators from mindfulness to symptoms such as FCR and fatigue in PWC. This is also the first study to test sex as a moderator of direct and indirect effects of the specified mediation models. This sample included an equal split of males and females and diversity in terms of disease and treatment characteristics as well as income, allowing for high generalizability of results and ultimately helping to address this gap.^1,65^ Results suggest that rumination may be more important than EA in accounting for the relationship between mindfulness and depression, anxiety, and FCR, especially for females.

### Rumination as Mediator

Rumination was a significant mediator from mindfulness to depression and anxiety, and to FCR, but not to fatigue. Consistent with expectations, rumination mediated the relationship between mindfulness and depression and anxiety indicating that mindfulness may work to reduce anxiety and depression through reducing rumination. This is consistent with evidence from experimental studies in cancer-populations showing MBIs reduce rumination,^66^ and that reduced rumination predicts lower distress,^67^ and mediates the effects of an MBI on depression^52^ and anxiety.^34^ This is also consistent with cross-sectional results showing rumination mediates the relationship between mindfulness and negative affect.^54^ Moreover, rumination mediated the relationship between mindfulness and FCR. This indicates that mindfulness may work to reduce FCR as well as generalized anxiety through reducing rumination, which is consistent with evidence showing MBIs reduce FCR and rumination.^68,69^ These results emphasize the importance of targeting rumination specifically in MBIs as a mechanism by which to improve anxiety, depression and FCR.

It is surprising, and contrary to our hypotheses, that rumination did not act as a significant mediator to fatigue, since fatigue is closely associated cross-sectionally with rumination and constructs mediated by rumination such as anxiety and depression.^1,17,22,25,44^ Additionally, systematic reviews and meta-analyses demonstrate that MBIs reduce fatigue, depression and anxiety, and rumination in PWC.^1,6,31,70^ Furthermore, previous intervention and cross-sectional studies report that rumination mediates the relationship from factors related to mindfulness, such as psychological resilience to fatigue.^66,71^

While associations between mindfulness, rumination, and fatigue exist, research in this area lacks strength and consistency compared to the evidence supporting rumination as a mediator between mindfulness and anxiety, depression, and FCR. To date, this study is the first to specifically test rumination as a mediator between mindfulness and fatigue in cancer populations. Results suggest rumination may be more important for FCR, depression, and anxiety, perhaps because these outcomes are characterized by maladaptive cognitive coping styles, whereas fatigue can be understood more physiologically than cognitively.^1,70^ Therefore, the relationship between mindfulness and fatigue may be better mediated through another pathway, such as sleep.^72^ Furthermore, fatigue clusters with pain and sleep more commonly than with anxiety and depression.^26,27^ Taken together, future studies can improve this area of research by assessing other potential mediators in the relationship between mindfulness and fatigue to better understand how mindfulness works to reduce fatigue and inform the development of interventions tailored to address fatigue in PWC, as rumination may not be the best target.

### Experiential Avoidance (EA) as Mediator

Contrary to our hypotheses, EA was not a significant mediator from mindfulness to depression and anxiety or fatigue, but it was weakly related to FCR. The lack of mediation to anxiety and depression is surprising considering previous studies demonstrate EA as a significant mediator between mindfulness and depression and anxiety in PWC both cross-sectionally,^53^ as well as longitudinally.^34^ The null indirect effects of EA to fatigue were also unexpected given studies showing EA and fatigue are associated with one another,^39^ and studies demonstrating interventions that incorporate mindfulness, such as Acceptance and Commitment Therapy (ACT), reduce both EA and fatigue.^32^ However, studies have yet to assess EA as a mediator from mindfulness to fatigue specifically. Our results suggest that EA may act as a *weak* mediator between mindfulness and FCR, and that the direct effect of mindfulness on FCR is predominant over the indirect effect through EA. This is consistent with previous research showing that mindfulness itself is a stronger predictor of outcomes in mindfulness training programs than EA.^73^

The clustering of anxiety and depression together and the sequence of change in mindfulness, rumination, and EA may explain why EA is a weaker predictor of outcomes than rumination or mindfulness in the current study and in studies of intervention effects. EA may be a significant mediator to depression and anxiety separately, but when their shared variance is being tested, at the expense of each construct’s unique variance, the indirect effects may no longer be strong.^74^ This suggests EA is more closely related to the unique variance in each construct as opposed to their shared variance, which may be accounted for by another factor, perhaps rumination, as suggested by our results. Hence, reducing EA may improve outcomes and act as a mechanism of MBIs, but rumination may be a stronger mechanism.

Moreover, although Labelle and colleagues (2015) found EA mediated the effects of MBCR, this did not occur until rumination improved *first*. Rumination may serve an avoidant function,^75^ and may serve as a form of cognitive avoidance which better captures the key mechanism that leads to changes in the broader construct of EA and, subsequently, psychosocial outcomes.^33,34^ Additionally, a single time-point may not provide sufficient information to detect the complex and dynamic relationships among these variables. Thus, future studies exploring whether and to what degree EA acts as a mediator from mindfulness to various psychosocial outcomes in PWC are justified and should take into account the timing and sequence of changes in mediating mechanisms.

### Sex as a Moderator

Interestingly, results revealed that sex was not a significant moderator of the models tested which is consistent with MBI studies that do not find statistically different effects for males and females.^28^ However, these results are surprising given the sex differences observed in levels of mindfulness, psychosocial outcomes, rumination and EA were in the expected directions. Importantly, the strength of relationships among variables did vary across sex when subgroup analyses were run suggesting that, while the effect may be weak, there may be subtle sex differences.

Results of subgroup analyses support the notion that rumination and EA may be more important for females than males in accounting for the relationship between mindfulness and psychosocial outcomes. Results of the present study indicate mindfulness may work to improve depression and anxiety through reducing rumination for both males and females. This is consistent with findings from other studies showing that mindfulness reduces rumination for both male and female PWC.^66,67^ Interestingly, our results indicate rumination may act as a full mediator to depression and anxiety in females, which is supported by previous research. For example, in a study by Labelle and colleagues (2010), rumination was evidenced to fully mediate the effects of an MBI on depression in a female-only sample. However, when a mixed sample underwent MBCR, rumination was a partial mediator of the intervention effect on later changes in mindfulness and ER constructs responsible for change in depression and anxiety.^34^ Therefore, rumination plays a larger role in the effects of mindfulness on depression and anxiety for females when compared to males.

Unexpectedly, the indirect effects through rumination and EA to FCR were not significant for males and females separately in this study. This may indicate that sex does alter the strength of relationships among variables because the indirect effects were significant when males and females were tested together. The differences across sex in direct and total effects as well as effect sizes suggest that rumination and EA may play a stronger role for females in the relationship between mindfulness and FCR. Overall, more research is needed to elucidate potential sex differences in the role of rumination on reducing depression and anxiety due to mindfulness, and to delineate mechanisms accounting for the effects of mindfulness on FCR across sex.

Taken together, results on sex differences are mixed, which is also true within the literature on MBIs for PWC.^76^ Rumination and EA might be particularly important mechanisms of mindfulness for females. However, the degree to which sex differences are statistically and clinically meaningful remains unclear. While subgroup analyses revealed subtle sex differences, these findings are exploratory and should be interpreted with caution, as they did not reach statistical significance in the formal moderation tests. Further research with larger sample sizes and causal designs is required to properly delineate the moderating effects of sex.

### Limitations

There are notable limitations to the current study. First, using a cross-sectional design limits the ability to draw causal conclusions and may also explain some of the null findings, particularly regarding the potential sequential relation among mediators. Although the current study aimed to inform intervention studies, cross-sectional results may differ substantially from the results of intervention studies. This is because baseline prevalence rates can vary and without assessing change through the effects of an intervention, the complex and dynamic relationships among variables may not be captured.^77^ It is also important to acknowledge that the constructs tested in this study are highly correlated and may be overlapping, which further supports the importance of leveraging causal and longitudinal research designs to better understand the temporal ordering or relations among similar constructs.^34^ Additionally, the MAAS is a unidimensional measure of mindfulness, limiting the ability for a more nuanced evaluation of how the different facets of mindfulness may influence symptoms and mediators differently. Furthermore, the current analyses did not control for disease characteristics and time since treatment. Time since treatment is evidenced to impact psychosocial symptom prevalence,^78^ and symptom prevalence is related to type and stage of disease. Treatment type is known to impact executive functioning,^79^ an important mechanism in ER processes.^48^ Given that treatment and disease characteristics may impact psychosocial symptoms and emotion regulation, it is important that future studies look to model these factors as moderators or covariates.

This study employed a conservative alpha cut-off which can be seen as a limitation, but also a strength. Although a conservative alpha level manages the type 1 error rate well and we are quite certain that statistically significant results are therefore robust, it risks an inflated type 2 error rate and the possibility of missing other important relationships that did not meet the strict alpha cutoff. Future research should explore the relationships tested in this study either without correcting for multiple testing by testing a single model, employing a larger sample, or by using less conservative corrections such as the Holm-Bonferroni, or Benjamin-Hochberg to assess whether results vary. Finally, as with any statistical testing, having a smaller sample size, particularly for subgroup analyses, limits sensitivity to capturing true effects in the data and is a limitation of the current study.

### Implications and Future Directions

Given the results and limitations of the current study, future studies should continue to test rumination and EA as mediators in the relationship between mindfulness and psychosocial outcomes in PWC as well as explore various potential moderators of the direct and indirect effects. Moreover, researchers may expand investigations of mediated effects to include a larger range of psychosocial symptoms in PWC such as fatigue, pain, quality of life, FCR, sleep and physical function. Additionally, future studies may benefit from comparing models that specify rumination and EA as simultaneous mediators with models that specify a sequential ordering from rumination to EA in order to confirm that rumination is first to change in MBIs. It is crucial that future studies pay particular attention to ensuring large and diverse samples. Future studies can also look to expand the exploration of moderators by including more demographic factors (eg, age, race/ethnicity, socio-economic status, marital status) and disease characteristics (eg, cancer stage, type, and treatment type) as these factors are also evidenced to influence mindfulness, ER, and psychosocial outcomes.

Results of this study can be influential in informing the development, testing, and implementation of MBIs in PWC. Given that rumination may be more important than EA, MBIs can specifically target rumination which, in turn, may improve EA and psychosocial outcomes. Specific facets of mindfulness may be more or less important for reducing rumination vs EA,^34^ and researchers and clinicians may use this information to develop, test, and implement targeted MBIs that focus on the particular facets responsible for reducing rumination. Importantly, while it is known that MBIs reduce a wide range of psychosocial symptoms in PWC, whether these symptoms all share the same mechanism of action is largely unknown.

The results of this study suggest that rumination-targeted MBIs can have a positive impact on the lives of PWC. Importantly, these results suggest rumination may *not* be an important mechanism accounting for the reduction in fatigue by MBIs which is an important distinction that requires further research to confirm. Results also suggest reducing rumination may be particularly important for females. This information can be invaluable in informing the individualization of MBIs to meet variable needs across groups of people. Although some may benefit from a mindfulness practice focused on reducing rumination, others may benefit from a mindfulness practice focused on another mechanism or outcome. While delineating differences based on sex can be helpful in informing how and whether different groups of people may have variable experiences with MBIs, it is important not to use this information to make overgeneralizations or create stereotypes.

Results can also inform PWC themselves. Having a better understanding of what may be contributing to their distress (ie, rumination) and how to manage it (ie, with mindfulness practice) can be empowering for PWC and contribute to improvements in quality of life. Additionally, the knowledge that different people may require different intervention strategies can inform PWC of the complexity of psychosocial concerns and the flexibility that may be required to address these concerns.

## Conclusions

This study was a novel investigation testing ER strategies (ie, rumination and EA) as mediators in the relationship between mindfulness and various psychosocial symptoms in PWC. This cross-sectional design has captured relationships among mindfulness, emotion regulation, and psychosocial symptoms that exist at baseline within the population sampled. Namely, results suggest that those who are more mindful, ruminate less, which may explain why they also have lower depression, anxiety and FCR. Results also suggest that rumination may be more important than EA in accounting for the relationship between mindfulness and depression, anxiety, and FCR especially for females. These findings can inform researchers and clinicians about the potential mechanisms and moderators of mindfulness within the context of precision cancer care. Delineating potential mechanisms and moderators of mindfulness cross-sectionally may point to how mindfulness training programs work (ie, mechanisms of action) and inform future studies in tailoring MBIs to target mechanisms hypothesized to be responsible for specific outcomes. Additionally, understanding how demographic factors may moderate relationships among mindfulness, ER, and psychosocial outcomes can inform clinicians as they flexibly navigate diverse needs across groups of individuals receiving MBIs. While these results are cross-sectional and in need of further research, findings from the present study enhance the MBI-cancer knowledgebase with the potential to benefit the lives of PWC.

## Supplemental Material


Supplemental Material - Mindfulness and Psychosocial Symptoms in People with Cancer: Testing Rumination and Experiential Avoidance as Mediators, and Sex as a Moderator
Supplemental Material for Mindfulness and Psychosocial Symptoms in People with Cancer: Testing Rumination and Experiential Avoidance as Mediators, and Sex as a Moderator by Hanna S. W. Conradi, MSc, Tina Nguyen, BSc, Oluwaseyi A. Lawal, PhD, and Linda E. Carlson, PhD in Global Advances in Integrative Medicine and Health.

## Data Availability

Data will be made available after recruitment for SEAMLESS trial ends.
