# Supplementary material for: Mindfulness and Psychosocial Symptoms in People with Cancer: Testing Rumination and Experiential Avoidance as Mediators, and Sex as a Moderator
Source: Glob Adv Integr Med Health. 2025 Aug 8;14:27536130251367051. doi: 10.1177/27536130251367051 (PMC12334834; doi:10.1177/27536130251367051)
Supplement: Supplemental Material - Mindfulness and Psychosocial Symptoms in People with Cancer: Testing Rumination and Experiential Avoidance as Mediators, and Sex as a Moderator [file sj-pdf-1-gam-10.1177_27536130251367051.pdf]

Supplemental Material

Link to OSF page: https://osf.io/9bmyu/

Table 1.

Model Fit Indices

Model Breakdown by Outcome	χ^2 (*df*)	CFI	TLI	RMSEA	SRMR
Recommended Values →	*P=>.05*	*<.90*	*<.90*	*>.08*	*>.08*
Depression & Anxiety Cluster					
Mindfulness → Rumination → DepAnx	**1.6 (1)**	**1.00**	**.99**	**.06**	**.01**
Mindfulness → EA → DepAnx	**1.3 (1)**	**1.00**	**.99**	**.05**	**.02**
Age * Mindfulness → Rumination → DepAnx	31.04(5)*	**.92**	.77	.20	.12
Sex * Mindfulness → Rumination → DepAnx	**4.16(3)**	**1.00**	**.98**	**.05**	**.02**
Fear of Cancer Recurrence					
Mindfulness → Rumination → FCR	-	**1.00**	**1.00**	**0.00**	**0.00**
Mindfulness → EA → FCR	-	**1.00**	**1.00**	**0.00**	**0.00**
Mindfulness → Rum & EA → FCR	15.74(2)*	**.90**	.70	.23	**.06**
Age * Mindfulness → Rumination → FCR	29.24(2)*	.84	.27	.32	.13
Age * Mindfulness → EA → FCR	29.24(2)*	.77	-.02	.32	.12
Sex * Mindfulness → Rumination → FCR	-	**1.00**	**1.00**	**0.00**	**0.00**
Sex * Mindfulness → EA → FCR	-	**1.00**	**1.00**	**0.00**	**0.00**
Fatigue					
Mindfulness → Rumination → Fatigue	-	**1.00**	**1.00**	**0.00**	**0.00**
Mindfulness → EA → Fatigue	-	**1.00**	**1.00**	**0.00**	**0.00**
Age * Mindfulness → Rumination → Fatigue	29.24(2)*	.81	.15	.32	.13
Sex * Mindfulness → Rumination → Fatigue	-	**1.00**	**1.00**	**0.00**	**0.00**

Note. Acceptable fit indices are in bold, CFI = Comparative Fit Index, Total N = 134, DepAnx = depression and anxiety cluster, EA = experiential avoidance, FCR = fear of cancer recurrence, RMSEA = Root Mean Square Error of Approximation, SRMR = Standardized Root Mean Squared Residual, TLI = Tucker-Lewis Index, , $\chi 2$ (*df*) = chi-square test and degrees of freedom test.

Figure 1.

Correlation Matrix Showing Pearson Correlations Among Variables

	Age	AAQ	FCRI	MAAS	RRQ	Fatigue	Anxiety	Depression
**Age**	1							
**AAQ**	-0.108	1						
**FCRI**	-0.417	0.367	1					
**MAAS**	0.427	-0.286	-0.586	1				
**RRQ**	-0.382	0.225	0.537	-0.584	1			
**Fatigue**	-0.299	0.186	0.432	-0.583	0.455	1		
**Anxiety**	-0.441	0.25	0.608	-0.639	0.637	0.562	1	
**Depression**	-0.331	0.126	0.503	-0.476	0.535	0.627	0.784	1

Note. AAQ = Acceptance and Action Questionnaire measures experiential avoidance, Anxiety, Depression, and Fatigue measured with PROMIS, FCR = Fear of Cancer Recurrence or Progression Inventory, MAAS = Mindful Attention and Awareness Scale, RRQ = Rumination and Reflection Questionnaire, Significant p-values (p = .003) are indicated by a double asterisk (**) when below or equal to .001 and a single asterisk when (*) when between .002 and .003, Total *n= 134.*

Table 2.

Model Paths and Estimates

**Model Breakdown by Outcome and Mediator / Moderator**	**Beta (SE)**	**95% *CI***
Depression & Anxiety through Rumination		**LL UL**
Mindfulness → DepAnx	**-2.21 (0.48)****	-3.18, -1.23
Mindfulness → Rumination	**-0.55 (0.07)****	-0.69, -0.41
Rumination → DepAnx	**2.36 (0.41)****	1.46, 3.04
Indirect (Mindfulness → Rumination → DepAnx)	**-1.29 (0.23)****	-1.72, -0.79
Total	**-3.50 (0.44)****	-4.23, -2.66
Sex as Moderator		
Mindfulness → DepAnx	**-4.55 (1.26)****	-6.84, -1.74
Mindfulness → Rumination	**-0.90 (0.20)****	-1.25, -0.52
Rumination → DepAnx	**2.32 (2.32)****	1.54, 3.23
Sex → DepAnx	-0.59 (0.83)	-2.25, 1.02
Sex → Rumination	**0. 45 (0.12)****	0.23, 0.71
Mindfulness*Sex → DepAnx	1.43 (0.82)	-2.27, 2.93
Mindfulness*Sex → Rumination	0.29 (0.13)	0.05, 0.52
Indirect Effect (Male)	**-1.41 (0.34)****	-2.08, -0.80
Indirect Effect (Female)	**-0.75 (0.23)****	-1.25, -0.31

Direct Effect (Male)	**-3.12 (0.58)****	-4.16, -1.88
Direct Effect (Female)	-1.70 (0.65)	-3.07, -0.44
Total Effect (Male)	**-4.53 (0.51)****	-5.37, -3.41
Total Effect (Female)	**-2.44 (0.71)****	-3.87, -1.06
Proportion of Mediated Effect (Male)	**0.31 (0.08)****	0.18, 0.49
Proportion of Mediated Effect (Female)	0.31 (0.11)	0.16, 0.60
Index	0.67 (0.34)	0.09, 1.41
Depression & Anxiety through Experiential Avoidance		
Mindfulness → DepAnx	**-3.37 (0.43)****	-4.19, -2.54
Mindfulness → EA	**-0.29 (0.09)****	-0.48, -0.13
EA → DepAnx	0.44 (0.41)	-0.41, 1.19
Indirect (Mindfulness → EA → DepAnx)	-0.13 (0.13)	-0.42, 0.11
Total	**-3.50 (0.43)****	-4.39, -2.63
Fear of Cancer Recurrence through Rumination		
Mindfulness → FCR	**-3.05 (0.69)****	-4.53, -1.75
Mindfulness → Rumination	**-0.55 (0.07)****	-0.68, -0.41
Rumination → FCR	**2.34 (0.68)****	0.91, 3.53
Indirect (Mindfulness → Rumination → FCR)	**-1.28 (0.38)****	-2.05, -0.52
Total	**-4.33 (0.57)****	-5.46, -3.25
Sex as Moderator		
Mindfulness → FCR	**-7.34 (1.80)****	-11.4, -3.55
Mindfulness → Rumination	**-0.90 (0.22)****	-1.32, -0.36
Rumination → FCR	**2.15 (0.72)***	0.59, 3.63
Sex → FCR	-0.54 (1.09)	-2.69, 1.51
Sex → Rumination	**0.45 (0.12)****	0.22, 0.71
Mindfulness*Sex → FCR	2.65 (1.03)	0.46, 4.86
Mindfulness*Sex → Rumination	0.29 (0.14)	-0.02, 0.55
Indirect Effect (Male)	-1.31 (0.48)	-2.32, -0.36
Indirect Effect (Female)	-0.69 (0.30)	-1.37, -0.12
Direct Effect (Male)	**-4.69 (0.90)****	-6.51, -2.82
Direct Effect (Female)	-2.05 (0.72)	-3.46, -0.61
Total Effect (Male)	**-6.00 (0.73)****	-7.75, -4.66
Total Effect (Female)	**-2.74 (0.76)****	-4.35, -1.26
Proportion of Mediated Effect (Male)	0.22 (0.09)	0.06, 0.40

Proportion of Mediated Effect (Female)	0.25 (0.12)	0.06, 0.54
Index	0.62 (0.38)	-0.05, 1.51
Fear of Cancer Recurrence through Experiential Avoidance		
Mindfulness → FCR	**-3.87 (0.53)****	-4.90, -2.77
Mindfulness → EA	**-0.29 (0.09)***	-0.46, -0.13
EA → FCR	**1.59 (0.48)****	0.62, 2.55
Indirect (Mindfulness → EA → FCR)	-0.46 (0.21)	-0.88, -0.11
Total	-4.33 (0.53)**	-5.23, -3.19
Sex as Moderator		
Mindfulness → FCR	**-8.42 (1.61)****	-12.10, -5.68
Mindfulness → EA	-0.56 (0.29)	-1.23, -0.08
EA → FCR	**1.51 (0.48)***	0.63, 2.52
Sex → FCR	0.67 (1.09)	-1.41, 2.89
Sex → EA	-0.16 (0.17)	-0.54, 0.20
Mindfulness*Sex → FCR	**3.03 (1.01)***	1.13, 5.44
Mindfulness*Sex → EA	0.15 (0.20)	-0.19, 0.62
Indirect Effect (Male)	-0.61 (0.26)	-1.19, -0.19
Indirect Effect (Female)	-0.38 (0.30)	-1.06, 0.12
Direct Effect (Male)	**-5.39 (0.75)****	-7.04, -3.99
Direct Effect (Female)	**-2.36 (0.76)***	-3.88, -0.73
Total Effect (Male)	**-6.00 (0.75)****	-7.62, -4.72
Total Effect (Female)	**-2.74 (0.76)****	-4.20, -1.04
Proportion of Mediated Effect (Male)	0.10 (0.04)	0.03, 0.21
Proportion of Mediated Effect (Female)	0.14 (0.13)	-0.05, 0.45
Index	0.23 (0.23)	-0.35, 1.01
Fatigue through Rumination		
Mindfulness → Fatigue	**-4.11 (0.80)****	-5.72, -2.35
Mindfulness → Rumination	**-0.55 (0.06)****	-0.67, -0.43
Rumination → Fatigue	1.58 (0.82)	-0.07, 3.27
Indirect (Mindfulness → Rumination → Fatigue)	-0.86 (0.47)	-1.86, 0.05
Total	**-4.98 (0.66)****	-6.14, -3.55
Sex as Moderator		

Mindfulness → Fatigue	**-6.19 (2.06)***	-10.61, -1.85
Mindfulness → Rumination	**-0.90 (0.20)****	-1.24, -0.41
Rumination → Fatigue	1.68 (0.81)	0.13, 3.32
Sex → Fatigue	-1.29 (1.16)	-3.66, 0.92
Sex → Rumination	**0.45 (0.13)****	0.21, 0.73
Mindfulness*Sex → Fatigue	1.21 (1.35)	-1.60, 4.12
Mindfulness*Sex → Rumination	0.29 (0.12)	0.03, 0.52
Indirect Effect (Male)	-1.02 (0.51)	-2.04, -0.06
Indirect Effect (Female)	-0.54 (0.31)	-1.29, -0.04
Direct Effect (Male)	**-4.98 (0.96)****	-7.29, -3.15
Direct Effect (Female)	**-3.77 (1.12)****	-5.76, -1.60
Total Effect (Male)	**-6.00 (0.88)****	-8.03, 4.43
Total Effect (Female)	**-4.31 (1.06)****	-6.23, -1.97
Proportion of Mediated Effect (Male)	0.17 (0.09)	0.01, 0.36
Proportion of Mediated Effect (Female)	0.13 (0.10)	0.01, 0.36
Index	0.48 (0.32)	-0.06, 1.18
Fatigue through Experiential Avoidance		
Mindfulness → Fatigue	**-4.93 (0.63)****	-6.15, -3.70
Mindfulness → EA	**-0.29 (0.08)****	-0.45, -0.13
EA → Fatigue	0.18 (0.62)	-1.03, 1.39
Indirect (Mindfulness → EA → Fatigue)	-0.05 (0.18)	-0.41, 0.30
Total	**-4.98 (0.60)****	-6.15, -3.80

Note. Beta = unstandardized coefficients, *CI* = confidence Interval, DepAnx = depression and anxiety cluster, EA = experiential avoidance, FCR = fear of cancer recurrence, LL = lower limit UL = upper limit, significant p-values (p = .003) are indicated by a double asterisk (**) when below or equal to .001 and a single asterisk when (*) when between .002 and .003, SE = standard error, Total N = 134.
